# Determination of heavy metals and their availability to plants in soil fertilized with different waste substances

**DOI:** 10.1007/s10661-018-6941-7

**Published:** 2018-09-03

**Authors:** Jadwiga Wierzbowska, Peter Kovačik, Stanisław Sienkiewicz, Sławomir Krzebietke, Teresa Bowszys

**Affiliations:** 1 Chair of Agricultural Chemistry and Environmental Protection, Faculty of Environmental Management and Agriculture, University of Warmia and Mazury in Olsztyn, 10 719 Olsztyn, Poland; 2 Department of Agrochemistry and Plant Nutrition, Faculty of Agrobiology and Food Resources, Slovak University of Agriculture in Nitra, 949 01 Nitra, Slovakia

**Keywords:** Composted sewage sludge, Municipal compost, Heavy metals, Soil, Waste organic materials

## Abstract

Field trials were conducted in 2004–2015, in Bałcyny, on haplic Luvisol formed out of light boulder clay. The experiment consisted of the following treatments: control (no fertilization), NPK, manure (FYM), dried pelleted sewage sludge (DPSS), composted sewage sludge (CSS), compost made from municipal sewage sludge and straw (SSCS), compost Dano made from unsorted household waste (CUHW), and compost produced from urban green waste (CUGW). Over a period of 12 years, 30 t DM/ha of each manure and composts were used, that is, 10 t DM/ha in each rotation of a crop rotation sequence. Nitrogen fertilization was kept on the same level on all experimental plots. Soil samples from the 0- to 20-cm horizon were collected after the third rotation crop, which was winter wheat harvested in 2015. It has been demonstrated that CUHW raised the soil total Cu content the highest, while the soil content of Zn was elevated the most by DPSS. The content of the remaining heavy metals (Pb, Ni, Cr, Mn, and Fe) increased as well, but to a lesser extent. The soil abundance of phytoavailable forms of copper improved even greater (from 75% when fertilized with CUGW or CSS, up to 124% when treated with CUHW). Soil content of soluble forms of such metals as Zn, Pb, Cr, Mn, and Fe changed less. The content of all analyzed heavy metals in soil (a form approximating the total content) was significantly positively correlated with the content of organic carbon (C-org.). This is the evidence for stronger adsorption of the above elements in soil richer in organic matter. On the other hand, the content of available forms of heavy metals depended more on the soil pH than on its content of C-org.

## Introduction

With continually growing amounts of municipal sewage sludge, the problem of how to recycle this waste gains an ever greater importance. Sewage sludge is a noxious but unavoidable by-product of wastewater and sewage treatment. According to data supplied by the Polish Central Statistical Office, 568 thousand tonnes DM of sewage sludge were generated in Poland in 2015, of which 107.5 thousand DM t were used in agriculture (Environment 2016). Sewage sludge is also employed for remediation of degraded land (19.2 thousand DM t) and cultivation of plants grown for composting (47.1 thousand DM t). In the same year, 10.9 million tonnes of municipal waste were collected, of which 16% was recycled by composting.

Sewage sludge and sewage sludge composts are a valuable source of organic matter and nutrients. One of the most rational ways of sewage sludge utilization, especially after the waste has been composted, is to apply it in agriculture (Bowszys et al. 2009; Wieczorek and Frączek 2013). In the study of Kluczka et al. (2017), it is written that soils in Poland are not contaminated with heavy metals. According to the authors, this is an argument for the optimistic thing on the use of sludge in agriculture. Sewage sludge and sewage sludge composts intended to be returned to the natural environment must meet numerous safety requirements so as not to cause pollution (Regulation of the Minister … 2015; Introduction of Fertilizers … n.d.). As civilization progresses, more and more waste is generated and its chemical composition changes. Depending on the origin, waste can contain considerable quantities of harmful substances, including heavy metals or PAHs, in addition to which it can be contaminated by microorganisms and parasites (Wieczorek and Frączek 2013; Milinovic et al. 2014). An assessment of the bioavailability of heavy metals based exclusively on their total content in sewage sludge may be insufficient to make an informed decision about its application in agriculture. The content of heavy metals in sewage sludge does not resolve unambiguously the question of their potential uptake by plants (Gawdzik and Gawdzik 2012; Fadiran et al. 2014; Tytła et al. 2016). A speciation analysis enables us to gain broader knowledge and understanding of the mobility and bioavailability of various fractions of metals and therefore substantiates a more rational decision to use sewage sludge for agricultural purposes. An in-depth and comprehensive insight into the ecological consequences of soil enrichment with sewage sludge composts requires basic and experimental studies, which will generate data for the development of rational and safe use of such products.

The aim of this study has been to assess the impact of sewage sludge and sewage sludge composts on the content of total and mobile forms of selected heavy metals in soil.

## Material and methods

A field experiment was conducted in 2004–2015, in Bałcyny (53° 35′ 49″ N 19° 51′ 20″ E), on haplic Luvisol formed out of light boulder clay (WRB 2015). The field trials comprised three crop rotation sequences: potato, spring barley, winter oilseed rape, winter wheat. Selected soil properties prior to the experiment are collated in Table 1.

**Table 1:** Characteristics of soil at the beginning of the experiment

Component		Unit	Content
C-organic		g/kg	7.63
N-total		g/kg	0.64
pH 1 mol KCl/dm^3^		–	5.40
Hh		mmol(+)/kg	27.7
Available forms	P	mg/kg	45.1
	Cu	mg/kg	1.47
	Zn	mg/kg	10.11
	Mn	mg/kg	104.50
	Pb	mg/kg	9.90
	Cr	mg/kg	0.61
	Ni	mg/kg	0.75
	Fe	mg/kg	1280

The experiment consisted of the following treatments: control (no fertilization), NPK, manure (FYM), dried pelleted sewage sludge (DPSS), composted sewage sludge (CSS), compost made from municipal sewage sludge and straw (SSCS), compost Dano made from unsorted household waste (CUHW), and compost produced from urban green waste (CUGW). Fertilization treatments during the experiment were composed of 30 t DM/ha of manure or composts, i.e., 10 t DM/ha applied after each rotation crop. Nitrogen fertilization was on the same level in all treatments, namely in the years when organic materials were applied, a dose of N up to the recommended level for the fertilized crop was supplemented with mineral fertilizers. In the remaining years, only mineral fertilization was applied. Soil samples from each plot (ten individual punctures from each plot were a pooled sample) were collected using Egner’s sampling stick, from the 0- to 20-cm soil layer after the third rotation was over, i.e., after harvesting winter wheat in 2015.

The chemical analyses of soil were performed with methods generally used in agricultural analytical chemistry. Soil reaction (pH) was determined potentiometrically in potassium chloride solution (1 mol KCl/dm^3^) and hydrolytic acidity (Hh) was determined using the Kappen method. Quantities approximating to total content of heavy metals in soil were extracted in a mixture of perchloric and nitric acids (Ostrowska et al. 1991), and the available forms were extracted in 1 mol HCl/dm^3^ (Karczewska and Kabała 2008). The content of heavy metals was determined with the atomic absorption spectrometric method (AAS) on a Shimadzu AA-6800 apparatus. The share of mobile forms of heavy metals to their total content in soil was calculated according to the formula shares of forms soluble in 1 mol HCl/dm^3^

$$ \%\mathrm{of}\ \mathrm{forms}\ \mathrm{soluble}\ \mathrm{in}\ 1\mathrm{mol}\ \mathrm{HCl}/{\mathrm{dm}}^3=\frac{\mathrm{content}\ \mathrm{of}\ \mathrm{forms}\ \mathrm{soluble}\ \mathrm{in}\ 1\mathrm{mol}\ \mathrm{HCl}}{\mathrm{total}\ \mathrm{content}\ \mathrm{of}\ \mathrm{heavy}\ \mathrm{metal}}\times 100 $$

The content of dry matter in organic materials was determined with the gravimetric (over drying and weighing) method (PN-EN 15934:2013-02E), while the content of organic carbon was determined after dry mineralization in a muffle furnace at 520 °C (PN-EN 15936:2013-02E) and that of total nitrogen with the Kjeldahl’s method after mineralization in concentrated sulfuric acid. The content of heavy metals in manure and composts was determined with the atomic absorption spectrometric (AAS) method on a Shimadzu AA-6800, after wet mineralization in a 4:1 mixture of nitric and chloric acids with added HCl. The determinations were completed by referring to certified material (Trace Metals-Sewage Sludge 4, Sigma-Aldrich RTC, Inc.), Table 2.

**Table 2:** The determinations referring to certified material (Trace Metals-Sewage Sludge 4, Sigma-Aldrich RTC, Inc.)

Value of determination	The content of heavy metals in Sewage Sludge 4						
	Cu	Pb	Ni	Cr	Zn	Mn	Fe
Certified value (mg/kg DM)	482 ± 50.4	154 ± 12.4	163 ± 13.5	289 ± 30.4	1240 ± 181	693 ± 108	20,100 ± 4390
Determination value (mg/kg DM)	455.5	153.2	160.8	280.9	1075.6	615.2	22,398
Precision of determination (%)	94.5	99.5	98.7	97.2	86.7	88.8	111.4

The results of chemical analyses were processed statistically in Statistica 12^®^. Verification of the significance of differences between the data was supported by the Tukey’s test at a level of significance equal *α* = 0.05. Cluster analysis was made by agglomerative clustering (single-element clusters), and the Euclidean distance served as a metric of disagreement.

## Results and discussion

The tested organic materials applied to fertilize soil differed not only in the content of dry matter and organic matter, but also in content of heavy metals (Table 3). Compared to manure (FYM), compost made from urban green waste (CUGW) contained less copper (Cu) and zinc (Zn) (by 7 and 40%, respectively), and all composts and sewage sludge were less abundant in manganese (Mn). Composts and sewage sludge, however, contained higher amounts of the remaining heavy metals than manure.

**Table 3:** Selected chemical characteristics of organic materials tested in the experiment

Component	Manure (FYM)	Dried pelleted sewage sludge (DPSS)	Composted sewage sludge (CSS)	Compost made from municipal sewage sludge and straw (SSCS)	Compost “Dano” made from unsorted household waste (CUHW)	Compost produced from urban green waste (CUGW)
g/kg DM						
DM	266.4	203,1	851.4	560.0	647.0	811.0
C-org.	231.2	388.9	428.8	170.7	152.9	65.9
C/N	13.1	7.0	6.7	8.4	15.3	6.6
P-tot.	1.0	10.4	8.0	4.4	2.9	3.2
mg/kg DM						
Cu	36.8	402.0	249.0	65.5	297.0	34.1
Zn	223.0	980.0	1360.0	295	831.0	133.0
Mn	334.0	228.0	300.5	210.6	273.6	326.8
Pb	5.50	15.8	20.3	24.9	178.0	29.1
Cr	10.20	56.90	33.20	15.3	53.7	19.8
Ni	6.64	28.0	36.6	17.7	35.2	15.5
Fe	3340	15,590	11,260	8050	16,200	5197

Immobile fractions of heavy metals in stabilized sewage sludge prevail over their mobile forms. Thickening, stabilization, and hygienization of sewage sludge decrease the mobility of these elements (increasing the content of dry matter during the consecutive processing stages). This limits the migration of heavy metals from sewage sludge to soil solution as well as their accumulation in living organisms (Tytła et al. 2016). Drying sewage sludge before its incorporation in soil typically decreases the leaching of Zn while increasing that of Cu (Milinovic et al. 2014). However, it should be borne in mind that heavy metals immobilized in the oxidizable fraction may be a potential threat to the soil’s zone of aeration (Gawdzik and Gawdzik 2012).

**Fig. 1:**
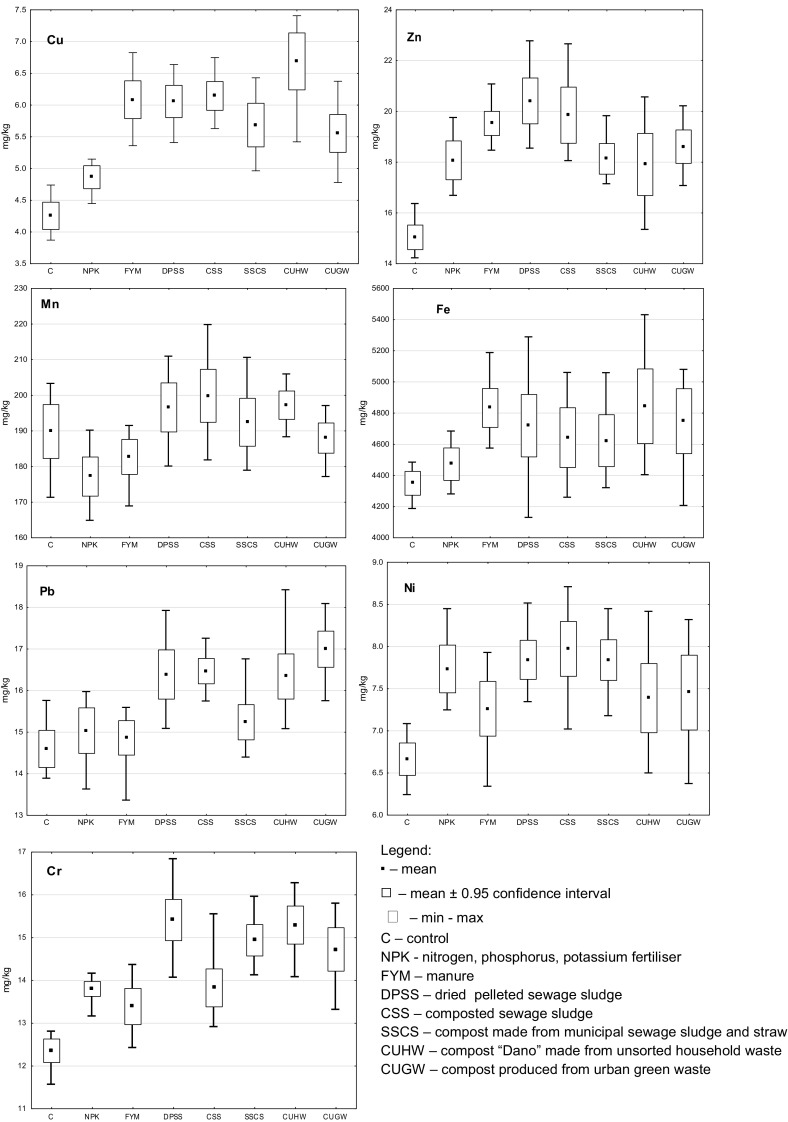
Content of heavy metals in soil (quantities approximating to the total content). Legend: ▪ mean, □ mean ± 0.95 confidence interval, min-max, C control, NPK nitrogen, phosphorus, potassium fertilizer, FYM manure, DPSS dried pelleted sewage sludge, CSS composted sewage sludge, SSCS compost made from municipal sewage sludge and straw, CUHW compost “Dano” made from unsorted household waste, CUGW compost produced from urban green waste

The content of heavy metals in the soil, as well as other elements, depends in the first place on the parent rock. The total content of the analyzed elements in Polish soils is quite strongly diversified and amounts to the following: Cu 4–36, Zn 30–360, Mn 50–1400, Pb 13–52, Ni 10–104, Cr 14–80, and Fe from 8000 to 27,800 mg/kg (Kabata-Pendias and Pendias 1999). Taking into consideration the above data, it should be stated that the content approximating to total content of heavy metals in analyzed soil did not exceed the range of natural content. The data presented in Fig. 1 show that, after the third crop rotation was terminated, the smallest quantities of the selected heavy metals, except Mn, were determined in soil of the control treatment (Cu 4.25, Zn 15.04, Mn 189.84, Fe 4349.74, Pb 14.60, Ni 6.66, and Cr 12.36 mg/kg). Fertilization with NPK mineral fertilizers, manure, and other organic materials tended to increase in the soil the quantities approximating to total content of heavy metals. Compared to the control treatment (total content of Cu and Zn, 4.25 and 15.04 mg/kg, respectively), the soil fertilized with compost from unsorted household waste (CUHW) had a content of Cu higher by 57%, and the soil treated with dried pelleted sewage sludge (DPSS) contained 35% more Zn. The content of the other heavy metals changed within a smaller range composted sewage sludge (CSS) favored the accumulation of total content of Mn (199.86 mg/kg), and compost produced from unsorted household waste (CUHW) and manure (FYM) increased the total content of Fe in soil (4843.8 and 4833.0 mg/kg, respectively). Among the elements which do not belong to plant nutrients, most Pb (17.00 mg/kg) was found in soil fertilized with CUGW (over 16% more than in the control soil), and Ni (7.97 mg/kg) was most abundant in soil fertilized with CSS (*ca* 20% than in control), while the soil fertilized with DPSS or CUHW was *ca* 25% richer in Cr (15.41 and 15.29 mg/kg, respectively) than in the control soil.

The content of all analyzed heavy metals in soil was significantly positively correlated with the organic carbon (C-org.) and phosphorus total (P-tot.) content (Table 4). The strongest correlation coefficients between C-org. contents were demonstrated with respect to the content of Cu (*r* = 0.74*), Cr (*r* = 0.63*), and Zn (*r* = 0.59*), while slightly weaker in relation to Fe (*r* = 0.45*), Pb (*r* = 0.42*), Ni (*r* = 0.31*), and Mn (0.29*). At the same time, the content of Cr (*r* = 0.43*), Cu (*r* = 0.35*), Ni (*r* = 0.29*), and Pb (*r* = 0.21*) was positively correlated with the content P-tot. in soil. Significant and positive correlation coefficients were also determined for the content of Cu (*r* = 0.57*), Fe (*r* = 0.47*), Zn (*r* = 0.33*), Cr (*r* = 0.24*), and Pb (*r* = 0.22*) versus the soil pH. The total content of Cr (*r* = 0.53*), Cu (*r* = 0.44*), Zn and Fe (*r* = 0.41*), and Ni (*r* = 0.40*) was positively correlated with the sorption complex capacity. Finally, the content of Cu (*r* = − 0.42*), Fe (*r* = − 0.37*), Pb (*r* = − 0.33*), and Cr (*r* = − 0.41*) was negatively correlated with the hydrolytic acidity of soil.

**Table 4:** Correlations coefficient *r* between selected soil properties and the content of heavy metals in soil (content approximating to total)

Selected soil properties	Content of heavy metals in soil						
	Cu	Zn	Mn	Fe	Pb	Ni	Cr
pH	0,57*	0.33*	0.07	0.47*	0.22*	− 0.02	0.24*
C-org.	0.74*	0.59*	0.29*	0.45*	0.42*	0.31*	0.63*
P-tot.	0.35*	0.13	0.10	0.10	0.21*	0.29*	0.43*
Hh	− 0.42*	− 0.08	− 0.09	− 0.37*	− 0.33*	− 0.05	− 0.41*
CEC	0.44*	0.41*	− 0.09	0.41*	0.13	0.40*	0.53*

*CEC* cation exchange capacity, *Hh* hydrolytic acidity

*Significant at *p* ≤ 0.05, *n* = 96

Presence of stabilized, insoluble organic matter in soil, containing high molecular humic acids, induces permanent binding of heavy metals and their immobilization. Low molecular organic substances, that is, organic acids, polyphenols, and fulvic acids, cause either complexing or chelating of heavy metals, and the resulting bonds are characterized by high mobility in the soil environment (Wu et al. 2003; Park et al. 2011).

**Fig. 2:**
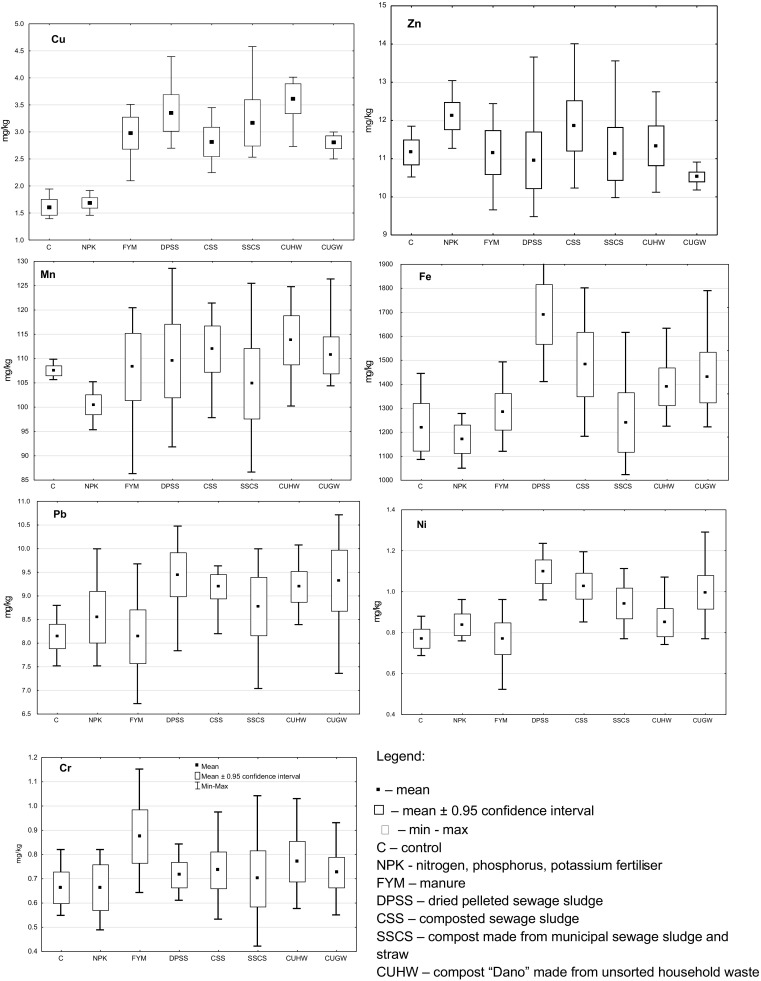
Soil content of heavy metals soluble in 1 mol HCl/dm^3^. Legend: ▪ mean, □ mean ± 0.95 confidence interval, min-max, C control, NPK nitrogen, phosphorus, potassium fertilizer, FYM manure, DPSS dried pelleted sewage sludge, CSS composted sewage sludge, SSCS compost made from municipal sewage sludge and straw, CUHW compost “Dano” made from unsorted household waste, CUGW compost produced from urban green waste

The contents of the determination heavy metals soluble in 1 mol/dm^3^ were found in the control soil in the following amounts: Cu 1.61, Zn 11.16, Mn 107.48, Fe 1220.55, Pb 8.14, Ni 0.77, Cr 0.66 mg/kg (Fig. 2). Similarly to its form approximating the total one, the least Cu soluble in 1 mol HCl/dm^3^ was contained in the control soil and in soil fertilized with mineral fertilizers alone. Organic substances used as soil fertilizers increased the content of the easily soluble form of this element (from 75% more in soil treated with CUGW and CSS, to 124% in soil fertilized with CUHW). The smallest amount of easily soluble Zn was determined in soil fertilized with CUGW (10.52 mg/kg), and the highest content of this Zn form was identified in soil fertilized with mineral NPK (12.11 mg/kg). The smallest amounts of easily soluble Mn and Fe forms were detected in the soil fertilized with mineral NPK (100.5 and 1170.9 mg/kg, respectively). The highest amount of easily soluble Mn was determined in soil fertilized with CUHW (113.8 mg/kg), while the highest amount of this Fe (1961.1 mg/kg) form was found in soil treated with DPSS (13 and 44% more, respectively, than in soil fertilized with NPK). The content of soluble forms of Pb and Ni in soil fertilized with manure was on the same level as in the control soil (8.14 and 0.77 mg/kg, respectively). Mineral fertilizers, composts, and, above all, dried pelleted sewage sludge (DPSS) contributed to an increase in the amounts of 1 mol HCl/dm^3^ soluble forms of Pb and Ni (by 16 and 44%, respectively). Organic fertilization raised the content of soluble Cr in soil, and the highest quantity of this metal (0.87 mg/kg) was detected in soil treated with manure (by about 32% more than in the control and NPK-fertilized soils).

The sequential analysis of the Zn and Cu content in sewage sludge showed that content of these metals in easily soluble and exchangeable fractions were low, and their highest amounts were bound in organic and residual fractions (Malinowska 2016a, b). After sewage sludge had been introduced to soil and the organic substance had been mineralized, the bioavailability of these metals increased, although it was still low regarding Cu, whose share of easily soluble and exchangeable fractions did not exceed 10% of the total content. According to Gondek (2010), fertilization with manure and sewage sludge did not cause significant mobilization of mobile Zn forms in soil in the first year. Due to the mineralization of organic matter and progressing acidification of soil, in the following years, the content of mobile forms of this element increased, but to a lesser extent than under the influence of mineral fertilization. In a study by Bowszys et al. (2015), sewage sludge compost significantly increased the soil content of available forms of Zn and Cu, but the increase was insufficient to change the soil’s classification to a higher category with respect to the abundance of these two elements. On the other hand, the content of Zn and Cu in soil leachate depended on the type of applied fertilizers. Most of Zn was leached from soil fertilized with composted sewage sludge, and most of Cu was leached from soil fertilized with composted sewage sludge and sewage sludge composted with straw.

Page et al. (2014) claim that soil reaction cannot be treated as a sole indicator of the mobility of heavy metals and their availability to plants, or the risk of their migration to the water environment. Tahervand and Jalali (2016) demonstrated that sorption and desorption of heavy metals in soil depended not only on the soil’s pH but also on its content of organic matter and calcium carbonate and on the chemical properties of a given metal. Cd and Ni are most strongly adsorbed in soil with a high content of organic matter, while Fe in soil rich in CaCO_3_. On the other hand, metals adsorbed by organic matter undergo desorption more easily than ones adsorbed by CaCO_3_, because sorption on carbonate minerals is often dominated by strong chemical reactions. Kopeć et al. (1991) suggest that organic fertilization leads to weaker penetration of trace elements deep into the soil profile. Another factor which influences the mobility of heavy metals is the maturity of compost added to soil. Fresh composts with a high content of low molecular organic acids may induce the mobilization of heavy metals present in both the applied compost and the soil (Zmora-Nahum et al. 2005; Weber et al. 2007). Sewage sludge added to soil cause changes in the soil mobility of heavy metals (Malinowska 2016a, b, 2017). As a result of the mineralization of organic matter, the mobility of heavy metals improves, whereas soil liming favors their binding in the residual fraction. According to Kuziemska et al. (2014), it is not only soil liming but also organic matter in the soil that are responsible for decreased mobility of nickel in soil. Mamindy-Pajany et al. (2013) claim that soluble organic carbon contained in sewage sludge applied to limestone soils can facilitate the leaching of Ni by creating soluble Ni-organic complexes. Moretti et al. (2016) showed that short-term application of sewage sludge and sewage sludge composts with low levels of heavy metals, tested in the tropical rainforest conditions, did not have negative effects on soil. The content of heavy metals remained below the maximum acceptable threshold values and approximated the geochemical background values reported for the research area. Sewage sludge composts introduced to soil cause changes in the soil mobility of metals. Sewage sludge composts led to a lower increase in the content of Cu and Ni in soil than sewage sludge did.

In this research, the content of the selected heavy metals soluble in 1 mol HCl/dm^3^, except Ni and Zn, was significantly positively correlated with the soil pH, whereas the soil content of C-org. left unaffected only the content of Zn in soil (Table 5). The content of Cu and Ni soluble in 1 mol HCl/dm^3^ was significantly positively correlated with P-tot. content in the soil (*r* = 0.44* and *r* = 0.23*, respectively). In turn, the content of soluble forms of Zn was negatively correlated (*r* = − 0.27*) with the content of P-tot. in the soil. A significant positive dependence was also confirmed between the capacity of the sorption complex and the soil’s content of easily soluble forms of Cu, Pb, and Cr. The content of Cu, Pb, and Mn soluble in 1 mol HCl/dm^3^ was significantly negatively correlated with the hydrolytic acidity of soil.

**Table 5:** Correlations coefficient *r* between selected soil properties and the soil content of heavy metals soluble in 1 mol HCl/dm^3^

Selected soil properties	Content of heavy metals in soil						
	Cu	Zn	Mn	Fe	Pb	Ni	Cr
pH	0.56*	0.11	0.22*	0.25*	0.40*	0.01	0.50*
C-org.	0.80*	− 0.09	0.37*	0.55*	0.50*	0.45*	0.23*
P-tot.	0.44*	− 0.27*	− 0.13	− 0.15	0.02	0.23*	− 0.16
Hh	− 0.46*	0.07	− 0.21*	− 0.05	− 0.31*	− 0.07	− 0.11
CEC	0.36*	0.10	0.01	0.16	0.36*	0.12	0.24*

*CEC* cation exchange capacity, *Hh* hydrolytic acidity

*Significant at *p* ≤ 0.05, n = 96

The content of heavy metals soluble in 1 mol HCl/dm^3^ also depended on the properties of organic materials used for soil fertilization (Table 6). The amount of easily soluble Cu was positively correlated with the C/N ratio (*r* = 0.30*) and the content of Cu in organic materials (*r* = 0.39*). In turn, the content of the mobile forms of Fe and Ni was positively correlated with the content of C-org. (*r* = 0.44* and *r* = 0.35*, respectively) and P-tot. (*r* = 0.43* and 0.58*, respectively). Also, the content of Fe in organic materials had a significant influence (*r* = 0.26*) on the amount of its easily soluble form in soil. The quantities of Fe, Pb, and Ni forms soluble in 1 mol HCl/dm^3^ were negatively correlated (*r* = − 0.31*, *r* = − 0.25*, and *r* = − 0.62*, respectively) with changes in the C/N ratio in organic materials, whereas the content of easily soluble Cr changed proportionally (*r* = 0.28*) to changes in the C/N ratio. The content in soil of mobile forms Pb was positively correlated (*r* = 0.26*), whereas the content of Cr easily soluble in soil was negatively correlated (*r* = − 0.24*) with the content of P-tot. in organic materials.

**Table 6:** Correlations coefficient *r* between the selected properties of organic materials and the soil’s content of heavy metals soluble in 1 mol HCl/dm^3^

Content of heavy metals in soil	Selected properties of organic materials									
	C-org	C/N	P-tot.	Cu	Zn	Mn	Fe	Pb	Ni	Cr
Cu	0.02	0.30*	− 0.08	0.39*						
Zn	0.21	0.09	0.21		− 0.06					
Mn	0.02	0.08	0.05			0.06				
Fe	0.44*	− 0.31*	0.43*				0.26*			
Pb	0.09	− 0.25*	0.26*					0.21		
Ni	0.35*	− 0.62*	0.58*						− 0.21	
Cr	− 0.04	0.28*	− 0.24*							0.01

*Significant at *p* ≤ 0.05, *n* = 72

According to Czarnowska and Kozanecka (2001), the content of soluble forms of Zn, Cu, and Cd in the soil’s humic horizon is highly significantly correlated with the total content of all forms of these metals in soil. The share of soluble forms in the total content of these metals decreased in the following order: Zn > Cd > Cu > Pb > Ni.

The data shown in Table 7 show that the shares of forms soluble in 1 mol HCl/dm^3^ in the total content of heavy metals were as follows: Zn (63.77%) > Pb (57.42%) > Mn (57.02%) > Cu (49.75%) > Fe > (29.67%) > Ni (12.32%) > Cr (5.27%). The smallest share of Cu soluble in 1 mol HCl/dm^3^ was determined in control soil and in soil fertilized exclusively with mineral fertilizers (37.57 and 39.97%, respectively). The organic materials added to soil, and particularly manure (FYM) and compost from unsorted municipal waste (CUHW), increased the share of soluble Cu forms. With respect to Zn, the highest share of this form of Zn was determined in the NPK-fertilized soil (80.90%), whereas manure (FYM), sewage sludge, and composts decreased the percentage of this form of Zn compared to soil fertilized with NPK mineral fertilizers and to the control soil. In the case of Mn, the share of easily soluble forms in its total content ranged from 53.07% in soil fertilized with NPK mineral fertilizers alone to 61.35% in soil fertilized with manure. The smallest share of easily soluble Fe was determined in soil fertilized with sewage sludge composted with straw (25.66%) and the highest one was identified in soil fertilized with DPSS (36.59%). The smallest solubility of Pb compounds (54.46%) was noted in soil fertilized with manure, while CSS significantly increased the share of soluble forms of this element. Similarly to Pb, the smallest share of soluble Ni forms was found in manure-treated soil. Sewage sludge and sewage sludge composts as well as compost made from urban green (CUGW) waste significantly raised the solubility of Ni compounds. Same as in the case of Cu and Mn, the highest share of Cr soluble forms was determined in soil fertilized with manure.

**Table 7:** Share of forms soluble in l mol HCl/dm^3^ in the total content of heavy metals in soil

Experimental object	Cu	Zn	Mn	Fe	Pb	Ni	Cr
	%						
Control (C)	37.57^a*^	74.51^d^	56.87^ab^	28.01^ab^	55.80^ab^	11.61^cd^	5.36^a^
NPK	39.97^a^	80.90^e^	53.07^b^	26.96^ab^	58.68^ab^	12.64^ad^	5.40^a^
Manure (FYM)	61.01^c^	61.89^bc^	61.35^c^	28.80^a^	54.46^a^	9.97^b^	6.34^b^
Dried pelleted sewage sludge (DPSS)	54.81^bc^	55.37^a^	55.07^ab^	36.59^d^	57.41^ab^	13.79^a^	5.19^a^
Composted sewage sludge (CSS)	50.36^b^	65.47^c^	58.30^a,c^	32.09^c^	60.47^b^	13.15^a^	4.90^a^
Compost made from municipal sewage sludge and straw (SSCS)	51.93^b^	56.89^a^	57.28^a-c^	25.66^b^	59.20^ab^	12.97^a^	5.27^a^
Compost “Dano” made from unsorted household waste (CUHW)	59.9^6c^	55.83^a^	58.08^a,c^	29.55^a,c^	56.29ab	10.87^bc^	4.98^a^
Compost produced from urban green waste (CUGW)	42.41^a^	59.33^ab^	56.12^ab^	29.66^a,c^	57.08^ab^	13.54^a^	4.74^a^

*Data designated with same letters do not differ significantly at *p* ≤ 0.05

According to Sienkiewicz and Czarnecka (2012), in alkaline soils, an increase in the content of soluble forms of Cu, Zn, and Mn in soil fertilized with large doses of sewage sludge (up to 280 t/ha), rather than being a threat to the environment, improved the nutrition of plants with these micronutrients. In another study by Sienkiewicz et al. (2009), it was demonstrated that regular manure fertilization increased the content of Cu, Zn, and Mn soluble forms compared to soil which received only mineral fertilization.

Nutrients applied in doses exceeding the requirements of plants lead to a change in the ionic equilibrium of the soil solution and to the leaching of these elements to groundwater (Gondek 2009). A single application of a dose of sewage sludge, even a large one, does not cause a distinct rise in the leaching of heavy metals from soil, when compared to the application of manure or even NPK mineral fertilization. However, due to the positive balance of these elements in soil, long-term use of sewage sludge may be problematic, especially when soil acidity increases, in the consequence of which the mobility of elements and their leachability rise (Sevel et al. 2014). In a study conducted by Wierzbowska et al. (2016), soil fertilization with organic waste did not pollute leachate water with Mn and Zn, while content of other metals rose to a level typical of unsatisfactory or even poor quality water. The content of Cu and Ni in leachate water depended on the quantities of other metals imported to soil with organic materials, for example, content of Cu and Ni decreased as the content of C-org. in fertilizers increased. Moreover, contents of Pb, Cr, and Mn in percolating water were positively correlated with the content of organic carbon on soil, and a higher C/N ratio contributed to the leaching Mn. According to Kalembasa and Pakuła (2009), incorporation of sewage sludge into soil increased the content of Zn, Cr, Ni, Pb, and Cu in the exchangeable fraction, reducing the fraction bound with Fe and Mn oxides and the oxidizing-organic fraction, while decreasing their content in the residual fraction. The shares of heavy metals in particular fractions largely depended on the content of organic carbon and the clay fraction, being less dependent on the total content of a given element in soil and soil sorption capacity.

**Fig. 3:**
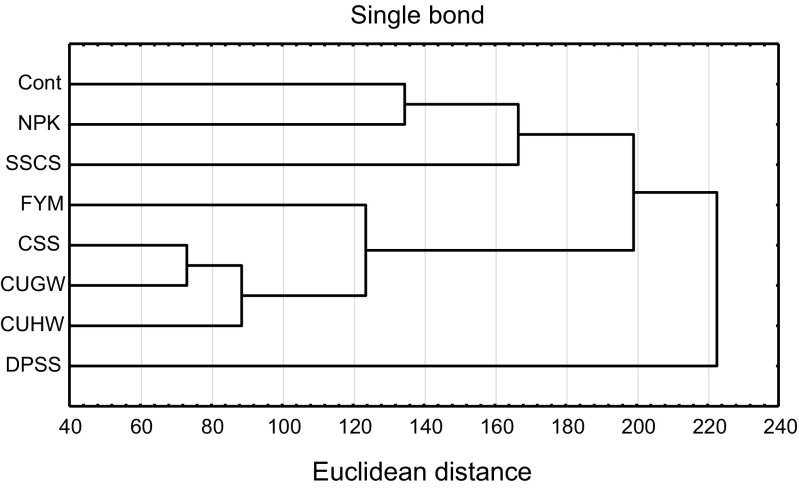
Dendrogram of the content of heavy metals in soil. C control; NPK nitrogen, phosphorus, potassium mineral fertilizer; FYM manure; DPSS dried pelleted sewage sludge; CSS composted sewage sludge; SSCS compost made from municipal sewage sludge and straw; CUHW compost “Dano” made from unsorted household waste; CUGW compost produced from urban green waste

Results of the cluster analysis enabled us to distinguish groups of fertilization objects with approximately the same content of heavy metals in soil (Fig. 3). The most similar content of these elements was found in soil fertilized with SSC and with CUGW. The content of these metals in soil fertilized with CUHW was only slightly different from their content in soil fertilized with SSC and CUGW. Another group consisted of control soil and NPK-fertilized soil. The content of heavy metals in soil fertilized with DPSS differed to the highest extent from their content in soil sampled from the other treatments.

## Conclusion

The experiment was conducted in a region free from the risk of soil pollution with heavy metals originating from industries. Moreover, the tested soil had developed from soil material poor in these elements. In compliance with the Polish law, the amounts of heavy metals observed in our study (forms close to the total content) were within the limits of their natural content in the superficial soil layer. It is therefore justifiable to claim that the tested waste substances and composts as well as NPK or manure applied in moderate doses do not create a risk of excessive accumulation of heavy metals in average soil. The content of available forms of trace metals, which are considered to be micronutrients owing to the roles they play in life processes (Cu, Zn, Mn, and Fe), was not high. On the contrary, the soil richness in micronutrients was within the range of unsatisfactory to medium. The content of all the analyzed heavy metals in soil (forms close to the total) was significantly positively correlated with the content of organic carbon (C-org.). In turn, the content of available forms of heavy metals was more strongly dependent on the soil pH than on its content of C-org. This set of relationships clearly demonstrates that even an elevated content of heavy metals in soil may not be a threat as long as an appropriate soil reaction is maintained.
